# One-step synthesis of ultra-long silver nanowires of over 100 μm and their application in flexible transparent conductive films

**DOI:** 10.1039/c7ra13683h

**Published:** 2018-02-20

**Authors:** Yuxiu Li, Shuailong Guo, Hongwei Yang, Yunxiu Chao, Shaozhuang Jiang, Chuan Wang

**Affiliations:** State Key Laboratory of Advanced Technologies for Comprehensive Utilization of Platinum Metals, Kunming Institute of Precious Metals 650106 Kunming People's Republic of China nanolab@ipm.com.cn

## Abstract

Silver nanowires (AgNWs) >100 μm and even 160 μm in length have been synthesized using a facile and rationally designed solvothermal method by heating preservation at 150 °C. The length of the as-synthesized AgNWs is over 4–5 times longer than those previously reported, while the diameter range is from 40 nm to 85 nm. A transparent conducting film (TCF) was fabricated using hydroxyethyl cellulose (HEC) as the adhesive polymer, and it achieved exceptional and stable optoelectronic properties. Its low sheet resistance of ∼19 Ω sq^−1^ (on polyethylene terephthalate, PET) and high optical transmittance of ∼88% are superior to that of expensive indium tin oxide (ITO) films. More significantly, the AgNW network demonstrates excellent adhesion to PET substrates. This study indicates that ultra-long silver nanowires can serve as an alternative to ITO, which also demonstrates its potential application in flexible electronic devices.

## Abstract 2

Ultra-long silver nanowires (100–160 μm) were applied in flexible transparent conductive films showing low sheet resistance and high optical transmittance.

## Graphical abstract

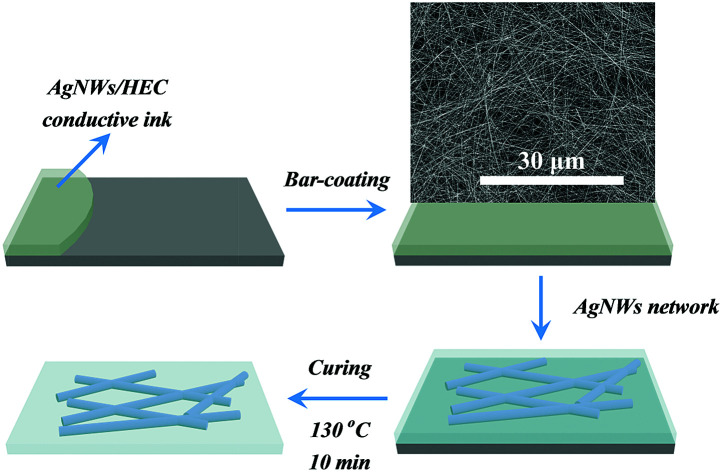

## 1. Introduction

Along with the rapid development of flexible electronic devices, such as flexible solar cells, flexible displays, organic light emitting diodes (OLEDs), and so on, the supply of and demand for flexible transparent conductive films (TCFs) is increasing. Future electronics will be non-planar, twisted and compressed shapes, and meanwhile possess excellent performances. The current research has been devoted to nano-materials for flexible transparent conductive films, such as graphene,^1,2^ carbon nanotubes (CNTs),^3,4^ metal nanowires,^5,6^ and conducting polymers.^7^ Among these nano-materials, metal nanowires, especially silver nanowires (AgNWs) have been regarded as the main candidate to replace indium tin oxide (ITO) due to their ultrahigh conductivity, large length-diameter ratio, and excellent flexibility. In spite of the many abovementioned advantages of AgNWs, they have also shown many limits in their applications in flexible transparent conducting materials,^8^ because the current widely used AgNWs are relatively short. The length range of the reported AgNWs is from 0.5 μm to 31.2 μm, which are summarized in Table 1.^6,9–23^ Some reports have proved that the performance of the AgNWs relies mainly on the nanowire structure, such as diameter, length, dispersity, and so on.^24,25^ More importantly, the lengths of AgNWs are key elements for forming the high transmittance with a low haze, and low sheet conductivity. This is due to longer AgNWs can form a more effective network with smaller nanowire number density. Hence, the synthesis of longer AgNWs becomes especially necessary and crucial for the development of flexible transparent conductive films.

**Table 1:** Comparison of the length of AgNW synthesized by various methods

Preparation methods	Length (μm)	Ref.
Two-step polyol reduction method	10–15	6
Polyol synthesis method	25	9
Polyol synthesis method	0.5	10
Polyol synthesis method	15	11
Polyol synthesis method	30	12
Polyol synthesis method	3–13	13
Polyol synthesis method	2–15	14
Polyol synthesis method	10	15
Polyol synthesis method	10	16
Polyol synthesis method	15	17
Polyol synthesis method	10	18
Polyol synthesis method	8.7	19
Polyol synthesis method	9–15	20
Solvothermal method	8.63–29.8	21
Solvothermal method	31.2	22
Solvothermal method	5–10	23
**Solvothermal method**	**100–160**	**This work**

In the past several years, the polyol synthesis method has been the most common synthetic route for preparing AgNWs, as shown in Table 1.^6,9–20^ In this method, the poly(vinyl pyrrolidone) (PVP) was used as end-capping reagent and ethylene glycol (EG) was used as solvent and reducing agent to reduce silver nitrate (AgNO_3_) into AgNWs. It is observed that the preparation technology has revealed many shortcomings, such as tough experimental conditions, complicated synthesis processes, and the growth of nanowires is very sensitive to the presence of impurities, and so on. Surprisingly, the synthesis mechanism of the solvothermal method is similar with polyol synthesis method. Meanwhile, compared with polyol synthesis method, the solvothermal method is more simple and easy to control, the cost is low and the industrialized production is easy to be realized. However, there are just several reports about solvothermal method, and fewer studies attempted to synthesize long AgNWs. Although Chen *et al.*^21^ claimed that the solvothermal process can achieve AgNWs, the nanowires length is in the range of 8.63 to 29.8 μm with a wide distribution. Recently, Fang *et al.*^22^ have experimentally shown that the AgNWs were prepared *via* a solvothermal method, but the nanowires were below 30 μm. Liu *et al.*^23^ have prepared AgNWs using this approach, with length ranging from 5–10 μm. Therefore, the search for suitable process conditions capable of controlling the length of AgNWs has become the represent general trend.

In this communication, a straightforward and facile solvothermal method is proposed to grow ultra-long AgNWs. Long silver nanowires of over 100 μm with an acceptable diameter of about 40–85 nm have been successfully prepared. The as-synthesized AgNWs have been applied to fabricate flexible transparent conductive film. The AgNWs/HEC composite film achieved a low sheet resistance of ∼19 Ω sq^−1^ and a high optical transmittance of ∼88%. More significantly, the AgNW network demonstrates excellent adhesion (ISO/ASTM: 0/5B) to PET substrate. Therefore, this method can be a useful approach to grow ultra-long AgNWs for various flexible electronic devices.

## 2. Experimental

### 2.1 Materials

All chemical reagents including silver nitrate (AgNO_3_, ≥99.8%), poly(vinyl pyrrolidone) (PVP, *M*_w_ = 1 300 000), sodium chloride (NaCl, ≥99.5%), glycerol (GL, ≥99.0%), hydroxyethyl cellulose (HEC, Dow-QP-100 MH), ethanol (C_2_H_5_OH, ≥99.0%) were purchased from commercial sources. All of which were of analytical grades, were used as received without any further purification. Deionized (DI) water was prepared by laboratory water purification system with a resistivity of not less than 18.2 ΩM.

### 2.2 Preparation of silver nanowires

For a typical synthesis of ultra-long silver nanowires, a one-pot reaction was employed to mix all compounds and solvents. Briefly speaking, 23.0 mmol of PVP was first added to 70 mL of glycerol and completely dissolved using magnetic stirring at 100 °C. Meanwhile, 3.0 mmol of AgNO_3_ was added to 10 mL of glycerol, 0.062 mmol of NaCl was added to 20 mL of glycerol using magnetic stirring at 50 °C. Complete dissolution was required to obtain a transparent and uniform solution. Subsequently, the glycerol solutions of AgNO_3_ and NaCl were dumped into the glycerol solution of PVP, respectively, and stirred for three or five minutes. The mixture was then immediately transferred into a Teflon-lined stainless steel autoclave of 200 mL capacity, and preheated at 150 °C to grow AgNWs for 5 h until the reaction was complete. After the autoclave was cooled down to room temperature, the free-standing AgNWs was collected by centrifugation of 4500 rpm for 10 min and washed alternately several times by deionized water and absolute ethyl ethanol. Finally, the obtained AgNWs were re-dispersed in deionized water for further use.

### 2.3 Preparation of water-soluble AgNWs conductive ink

The AgNWs conductive ink was carried out by using HEC as adhesive. This process consisted of three steps: (1) the 1 wt% HEC aqueous solution was prepared by dissolving 0.04 g HEC in 3.96 g of deionized water. (2) 11 mL of the aqueous AgNWs dispersion solution, with the solid content of 0.72% was dispersed in above-mentioned solution by slow stirring. (3) 5 mL of the deionized water was further added under magnetic stirring to obtain conductive ink.

### 2.4 Preparation of AgNWs layer and thermal treatment

**Fig. 1:**
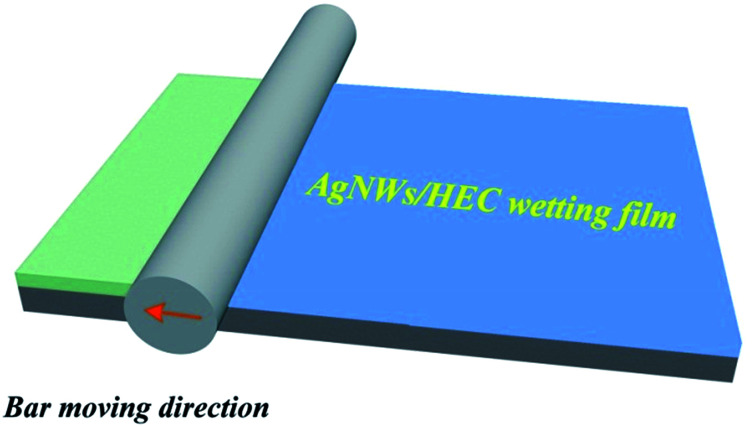
Schematic illustration of the bar-coating process.

**Fig. 2:**
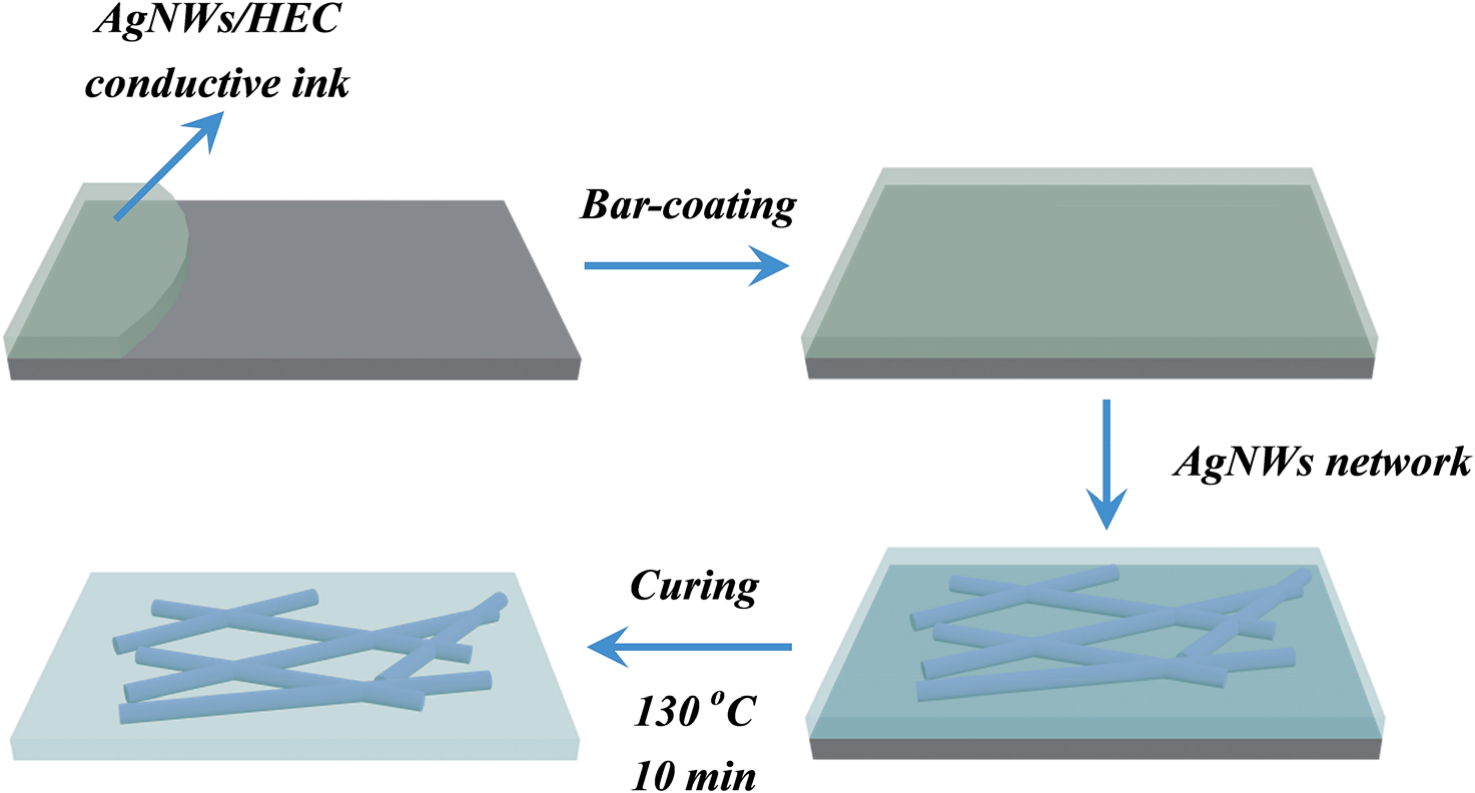
Schematic illustration of the fabrication procedure of AgNWs/HEC transparent conductive film.

The AgNWs conductive ink was used to fabricate a transparent conductive film using a straightforward bar coating process. More specifically, the amounts of AgNWs/HEC precursor solution was controlled precisely by dropper and transferred onto the PET substrate. Subsequently, the mixture was coated on the PET substrate using automatic film applicator (BEVS1811/2) with the bar (a diameter of 20 μm) at a bar speed of 300 mm s^−1^ (the bar-coating process was shown in Fig. 1), followed by curing at 130 °C for 10 min, and then formed an AgNW network on the PET substrate. Fig. 2 illustrates the fabrication procedure of AgNWs/HEC transparent conductive film.

### 2.5 Characterization of as-prepared samples

The structural characterization of the as-synthesized AgNWs was performed using X-ray diffraction (XRD: Rigaku TTRIII) on an X-ray diffractometer with Cu Kα radiation (*λ* = 1.54056 Å) operated at 40 kV to 200 mA in a scanning range of 10° to 90° (2*θ*). The size and surface morphologies of AgNWs were characterized by field emission scanning electron microscopy (FE-SEM), a few of the as-synthesized AgNWs were dispersed in absolute ethyl ethanol, and then was dropped in silicon wafer, the next step is to stuck it on conducting resin. The surface morphology of the as-fabricated AgNWs/HEC composite film were investigated by optical metallographic microscope (OM, NMM-800TRF) with an enlargement factor of 10 × 50 operating at a light intensity of 4, and the OM photograph was photographed through dark filed illumination. UV-vis spectrophotometer (PERSEE Genera TU-1901) was used to monitor the UV-vis spectra of the as-synthesized AgNWs. The transmittance and haze of the AgNWs/HEC composite film were measured with transmittance and haze analyzer (SGW-820) with a bare PET film as a reference. The technical parameters meet GB/T2410-2008 ASTM D 1003-61 (2007), such as JISK7105-81 test standards. The sheet resistance (*R*_sheet_) of the composite film was measured with a four point probe (SB100A/2). A tape was also used to test the adhesion of AgNWs/HEC conductive ink on the PET substrate by pressing the tape in painting has hundred grid ink surface, 45 degree angle quickly peeling off from the film.

## 3. Results and discussion

**Fig. 3:**
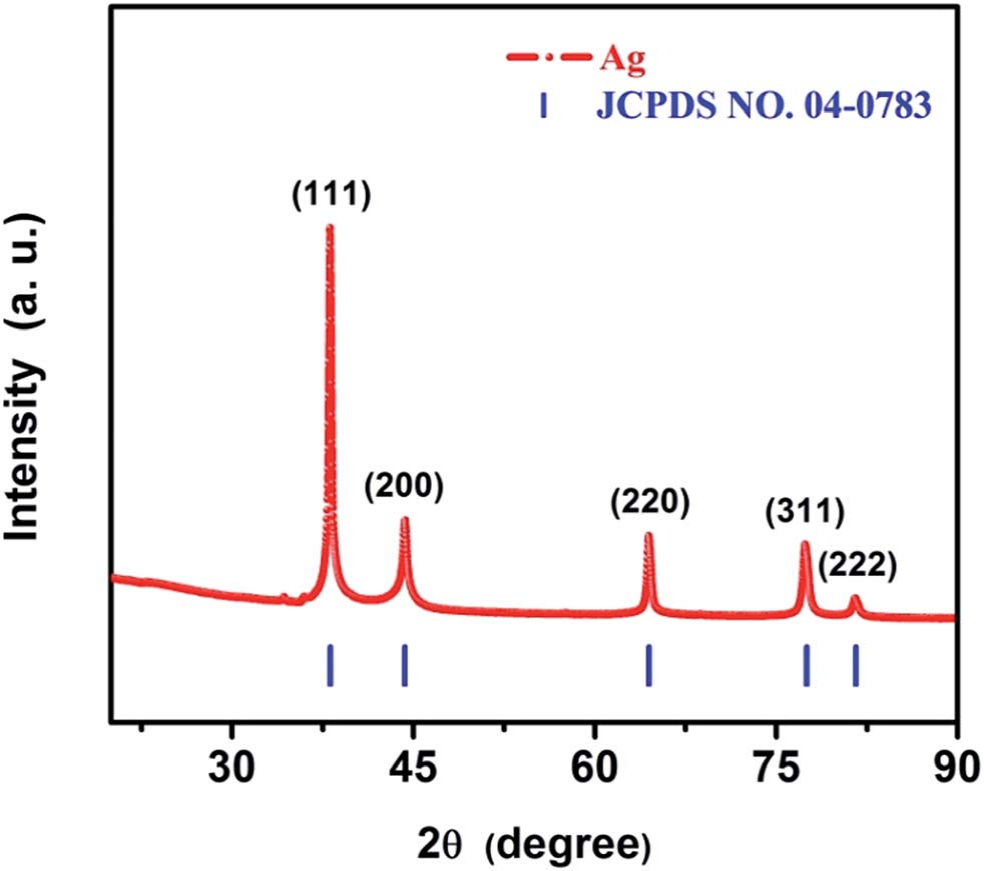
Typical XRD pattern of the as-synthesized AgNWs.

**Fig. 4:**
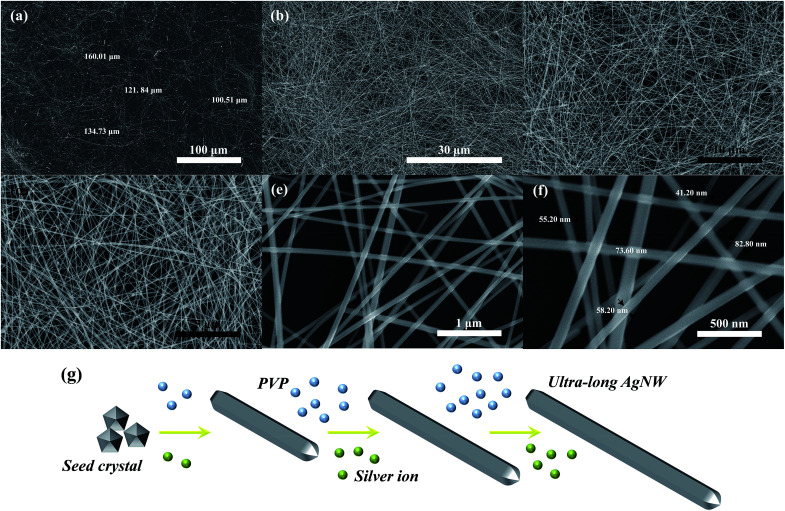
FE-SEM images of the as-synthesized AgNWs at (a, b) low magnification, (c, d) relatively high magnification and (e, f) high magnification. (g) Schematic of the proposed growth mechanism of AgNWs.

The phase purity and crystalline structure of the as-synthesized AgNWs were first characterized by X-ray techniques. Fig. 3 shows typical diffraction patterns of the as-synthesized product. All the diffraction peaks can be indexed to the face centered cubic (fcc) Ag crystal (JCPDS card no. 04-0783, *a* = *b* = *c* = 4.086 Å, space group *Fm*3̄*m* (225)). The peaks centered at 38.12°, 44.28°, 64.43°, 77.47° and 81.53° correspond to the (111), (200), (220), (311) and (222) reflections of Ag, respectively. Interestingly, no other characteristic peaks were observed such as Ag_2_O in the XRD pattern of as-synthesized product, implying the formation of AgNWs. Meanwhile, the crystal sizes of AgNWs were calculated from the peaks in the XRD pattern is 22 nm in the (111) direction, 14 nm in the (200) direction using Scherrer equation *d* = 0.943*λ*/*β* cos *θ* (*λ*, wavelength of the X-rays; *β*, full width at half-maximum; *θ*, angle of the diffraction). These crystal sizes are smaller than the smallest diameter (40 nm) of the nanowires (as shown in Fig. 4(f)), which indicates that the AgNWs are polycrystalline. Moreover, the sharp and strong peaks suggest that the as-synthesized AgNWs possess excellent crystalline degree.

The nano-morphology of the as-synthesized AgNWs was observed by FE-SEM and images are shown in Fig. 4. Fig. 4(a, b) display the macrographic FE-SEM images of as-synthesized AgNWs, from which it is observed that the high purity AgNWs were successfully synthesized with a long length of 100–160 μm using the solvothermal method. The magnified images displayed in Fig. 4(c, d), as can be seen from both images, no other silver nanostructures, such as spheres, cubes and polyhedron were observed. The more detailed structural features of as-synthesized AgNWs were examined by high magnification FE-SEM images in Fig. 4(e, f). It can be noticed that the nanowires with a diameter in the range of 40–85 nm. In addition, Fig. 4(g) presents the proposed schematic growth mechanism of ultra-long AgNWs in this work, which is similar to the nanowires synthesized in the seed-mediated approaches. At the beginning of the reaction, Ag seed crystals of pentagonal cross section with *D*_5h_ symmetry are firstly formed, and then their diameter increase *via* homogeneous and heterogeneous nucleation. Afterwards, these Ag seed crystals tend to form AgNWs.^26^ Under hydrothermal conditions, the growth process of AgNWs requires a completely stationary solution environment and a sufficient growth temperature. The AgNWs will exhibit spontaneous growth and free assembly after the seeds of AgNWs have been formed in the nucleation stage.^27^ In addition, long nanowires will be obtained when the molecular weight of PVP big enough, this is because the higher molecular weight of PVP, the longer molecular chain of PVP, and thus get longer AgNWs.^28^ Based on the above analysis, the temperature and organic capping agent are crucial to determine the length of nanowires on the hydrothermal process. In this work, firstly, the relative low reaction temperature of 150 °C in glycerol system and the condition of no-agitation have provided a suitable and effective growth environment for the formation of ultra-long AgNWs. Secondly, the PVP with molecular weight of 1 300 000 was used as blocking agent in this research, which should also contribute to the formation of ultra-long AgNWs. On the other hand, the plenty of reaction temperature of 5 h and the appropriate pressure of about 0.3 MPa will help Ag^+^ is reduced to Ag^0^ as much as possible in the later growth stage, and directly lead to the formation of ultra-long AgNWs.

**Fig. 5:**
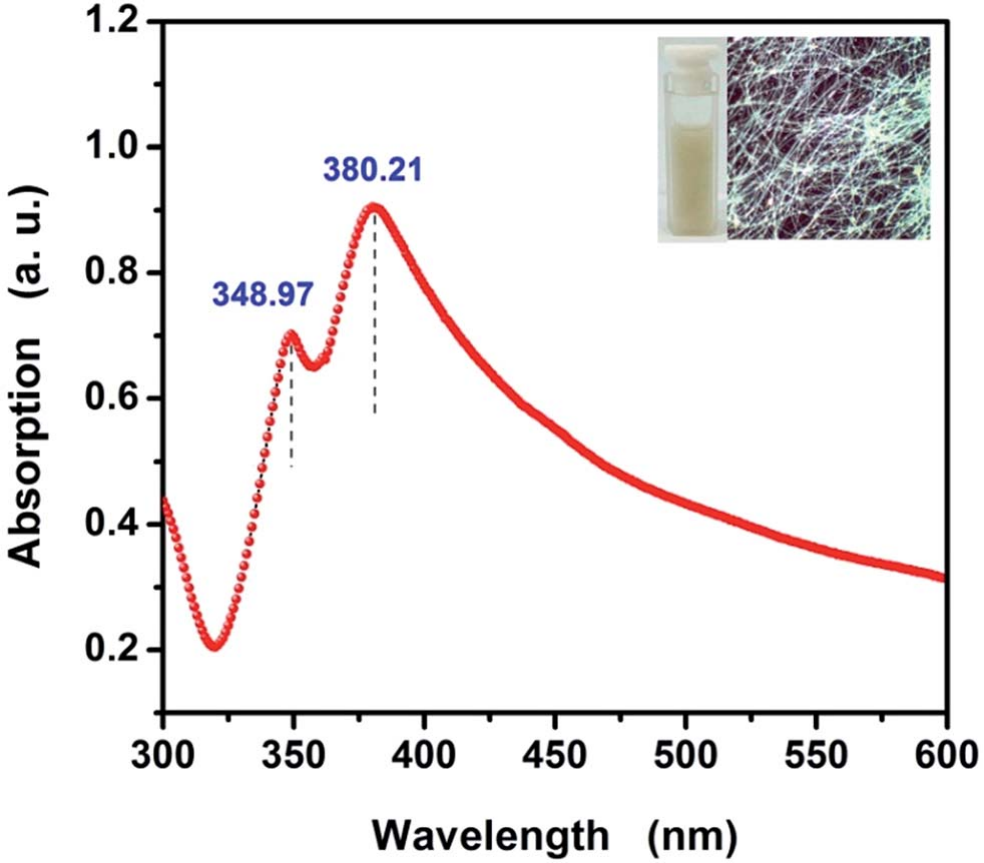
UV-vis absorption spectrum of the as-synthesized AgNWs suspension. The inset shows the photograph of the AgNWs suspension and the OM photograph of the as-fabricated AgNWs/HEC composite film.

The UV-vis absorption spectrum of the as-synthesized AgNWs suspension is shown in Fig. 5. UV-vis spectroscopy can be used to track the morphology of AgNWs because Ag nanostructures having different shapes exhibit surface plasmon resonance (SPR) bands at different frequencies.^29^ The UV-vis spectrum displays two obvious SPR peaks at 380.21 nm and 348.97 nm, respectively. The peak at 380.21 nm is due to the transverse plasmon resonance absorption. The other one is due to the longitudinal plasmon resonance absorption with the peak at 348.97 nm. The two obvious SPR peaks are regarded as the optical signatures of AgNWs.^30,31^ Most notably, no other absorption peaks were observed after 400 nm in the UV-vis spectroscopy of the as-synthesized AgNWs. This means that the as-obtained AgNWs basically without other silver nanostructures or silver nano-particles. This is consistent with the results of FE-SEM observations. The inset shows the photograph of the aqueous AgNWs dispersion solution and the optical metallographic (OM) photograph of as-fabricated AgNWs/HEC composite film, from which we can observe the AgNWs suspension appeared to be yellow white color, it is a typical colour of AgNWs dispersion. The OM photograph indicates that the AgNWs formed a uniform network without significant nanowire density differences across the PET substrate. The AgNWs network possesses numerous crossover junctions, which can provide efficient electron transfer routes for electrical conduction.

**Fig. 6:**
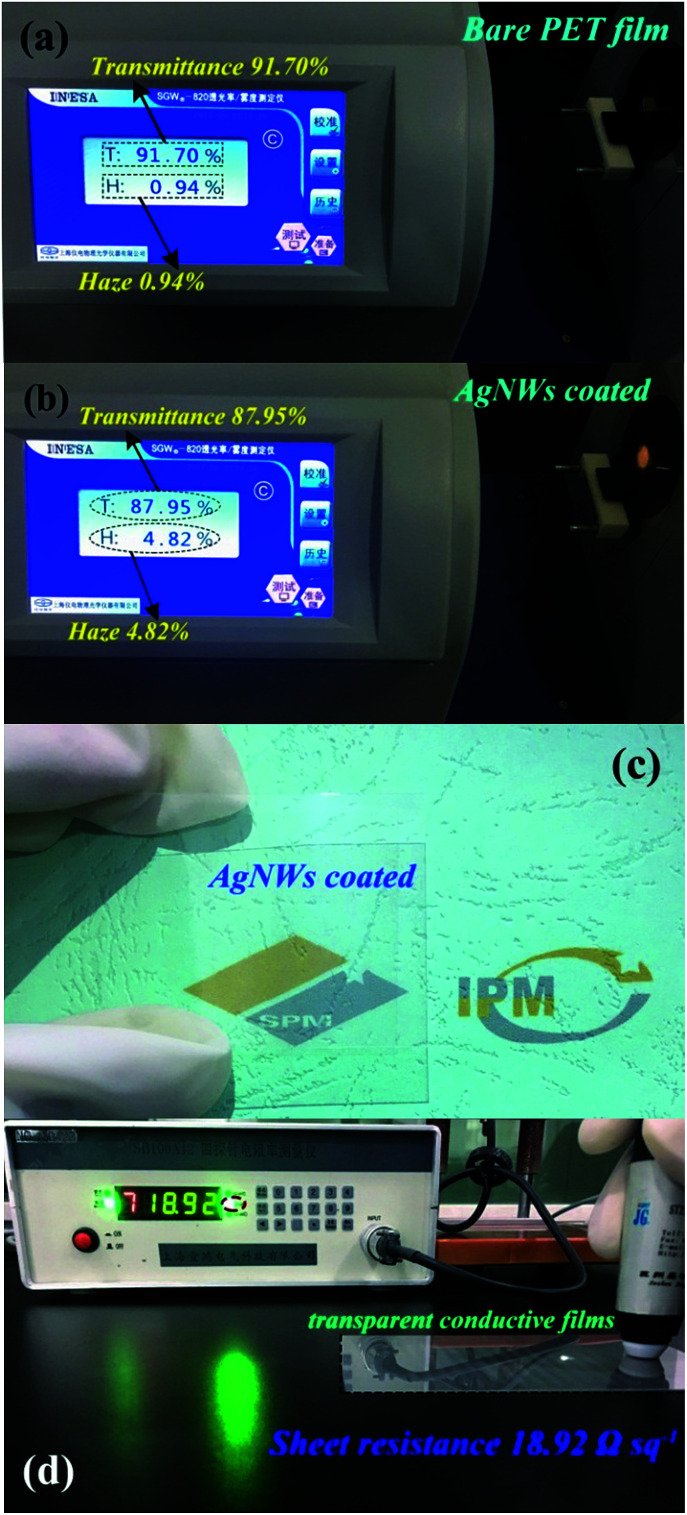
(a) The transmittance and haze based on the bare PET substrate. (b) The transmittance and haze based on the as-fabricated AgNWs/HEC composite film. (c) Optical photograph for demonstration of the AgNWs/HEC composite film with higher transmittance. (d) Sheet resistance measurement of the AgNWs/HEC composite film.

To wonder whether the as-synthesized AgNWs can serve as an alternative to the ITO for flexible transparent conductive film with higher transmittance and lower sheet resistance, we tested the transmittance/haze and sheet resistance of the AgNWs/HEC composite film, as shown in Fig. 6. The AgNWs/HEC composite film exhibited superior optical transparency and electrical conductivity. Fig. 6(a) clearly illustrates the test result of the transmittance and haze for bare PET substrate, the transmittance and haze are 91.70% and 0.94%, respectively. When the AgNWs conductive ink (AgNWs/HEC compound) was coated on the PET substrate, the transmittance mildly reduces from 91.70% to 87.95%, the haze slightly increases from 0.94% to 4.82% (Fig. 6(b)). The AgNWs/HEC composite film was a bit hazy due to substantial light scattering from the AgNWs. Fig. 6(c) presents a typical photograph of an AgNWs/HEC composite network on a PET substrate. The photograph shows that the AgNWs conductive ink was uniformly coated on the PET substrate, and the composite film is sufficiently uniform and transparent, and the logo below the film could be seen clearly. Furthermore, the sheet resistance of the composite film was further evaluated, as shown in Fig. 6(d). The sheet resistance of the AgNWs/HEC composite film is ∼19 Ω sq^−1^. To the best of our knowledge, this is a higher transparency and a lower sheet resistance among the values reported previously for transparent conductive AgNWs films.^32–35^ A Kapton tape was also used to test the adhesion of AgNWs/HEC conductive ink on the PET substrate, and the result indicates that the AgNWs/HEC conductive ink possesses an adhesion of 0/5B (ISO/ASTM grade) on the PET substrate. The adhesion is very strong and excellent for transparent conductive films without any extra treatments.

**Fig. 7:**
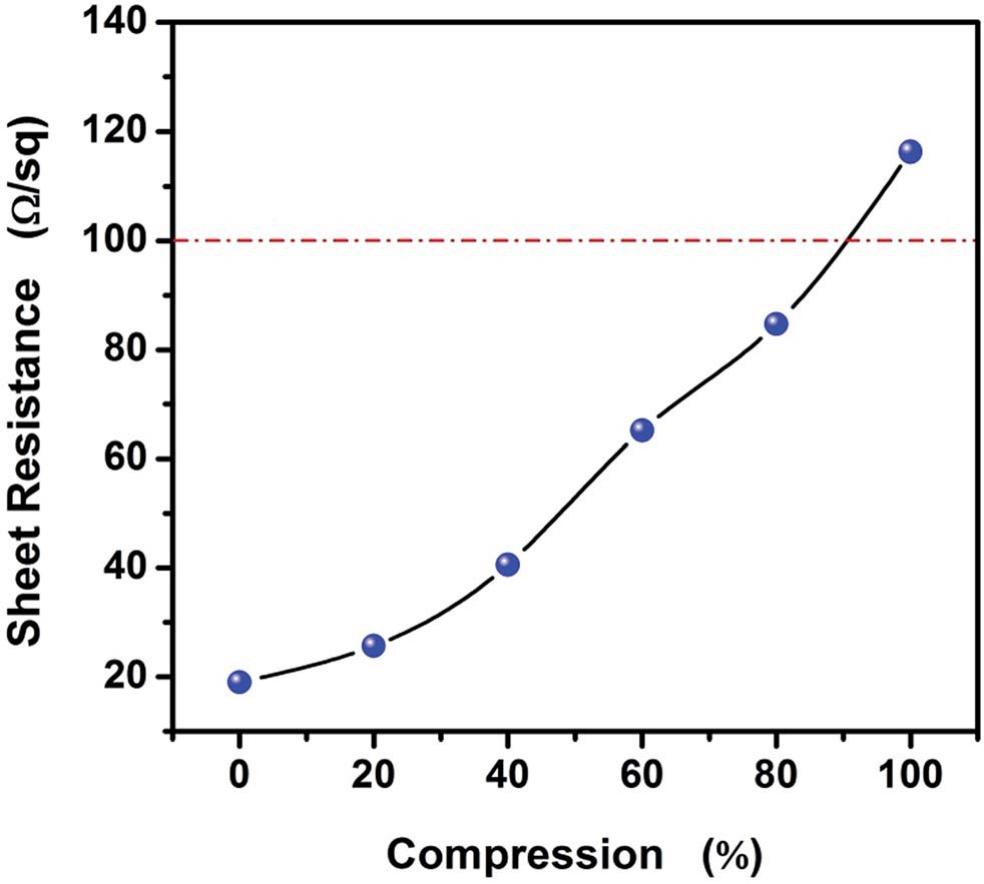
Variation of sheet resistance with compression strain for AgNWs/HEC composite film.

To study the variety of sheet resistance with strain for AgNWs/HEC composite film, Fig. 7 show the variation in sheet resistance in the compression process for AgNWs/HEC composite film. It can be observed that the larger compression degree of AgNWs/HEC composite film, the larger sheet resistance. And, more remarkable, the AgNWs/HEC composite film can have low sheet resistance (∼116 Ω sq^−1^) even at 100% strain. It is worth noting that the variety of sheet resistance is much shorter than that of other reports.^36^

## 4. Conclusion

In summary, this work demonstrated a fast, straightforward and facile method for growing ultra-long AgNWs. The diameters range of these AgNWs from 40 nm to 85 nm with a length of 100–160 μm which is fivefold as long as the AgNWs normally used. The ultra-long AgNWs have been used to fabricate transparent conductive film using HEC as an adhesive polymer *via* a simple bar coating technique, which achieved low sheet resistance of ∼19 Ω sq^−1^, high transmittance of 88%, and strong adhesion of 0/5B (ISO/ASTM grade), and can accommodate significant strains under extreme compression conditions. We anticipate that the AgNWs/HEC composite film can be used as an alternative to ITO film for emerging optoelectronic devices.

## Conflicts of interest

There are no conflicts to declare.

## Supplementary Material
